# The Influence of Selected Winter Wheat Cultivars Grown in Poland and the Growing Season on the Quality of Wheat Beers Produced from Them

**DOI:** 10.3390/foods15030601

**Published:** 2026-02-06

**Authors:** Justyna Belcar, Józef Gorzelany

**Affiliations:** 1 Farming Cooperative SAN, Łąka 598, 36-004 Łąka, Poland; 2 Department of Food and Agriculture Production Engineering, University of Rzeszów, St. Zelwerowicza 4, 35-601 Rzeszów, Poland; jgorzelany@ur.edu.pl

**Keywords:** wheat cultivars, beer quality, extractivity, attenuation, bitterness, wheat beers

## Abstract

The wheat cultivar significantly influences the quality of grain and malt, which are used to produce wheat beers, determining their potential for use in brewing. The brewing process influenced the quality of wheat beers obtained from selected winter wheat cultivars (Elixer, Lawina, Gimantis and Rockefeller cultivars) from three growing seasons. Wheat beers obtained from malt from the Rockefeller and Gimantis cultivars were characterized by the highest real extract values, ethyl alcohol content (4.14 and 4.05% *v*/*v*, respectively), and therefore the caloric content of the finished beer product (the lowest energy value was obtained for the Lawina cultivar—43.19 kcal·100 mL^−1^). Furthermore, wheat beers obtained from malt from the Gimantis cultivar were characterized by the lightest color and the lowest bitterness perception (14.8 IBU). The highest quality wheat beers were obtained with grain from the second growing season, regardless of the cultivar used. Cluster analysis of all quality characteristics of wheat beers obtained from malt derived from winter wheat grain grown in field experiments showed that the Gimantis cultivar, and to a lesser extent the Rockefeller cultivar, was characterized by the lowest quality variability among wheat beers brewed from malt derived from cereals from three growing seasons in the Lower Silesian Voivodeship.

## 1. Introduction

Beer quality characteristics vary depending on the style of beer brewed. The most important include apparent and real extract, real degree of attenuation, alcohol content, color, pH and acidity, carbon dioxide content, and bitterness. Wheat beers should be characterized by an abundant, long-lasting head, which can also be analyzed. The flavor and aroma qualities of wheat beers and their alleged health benefits can be determined by measuring volatile organic compounds, total polyphenol content, polyphenol profile, and antioxidant activity [1].

Extractivity refers to the chemical compounds transferred to the wort (from the malts) during mashing. Apparent extract of beer indicates the concentration of soluble compounds remaining in the beer after fermentation and can be measured with a hydrometer [1]. Real extract is the content of chemical compounds after distilling alcohol from beer [2]. The main components of real extract are carbohydrates (mainly dextrins, e.g., maltopentose), and in smaller amounts proteins (approx. 6–9%), glycerol, non-starch polysaccharides, organic and bitter acids, minerals, and many others [2].

The real degree of attenuation of beers significantly affects the ethyl alcohol level, which is one of the most important quality characteristics, as it is responsible for the sensory impressions of the produced beer, as well as for the economics of production, and is the value based on which tax is determined [3]. The degree of fermentation is important in beer brewing technology because it determines the efficiency of alcohol and carbon dioxide production using a properly selected yeast strain; therefore, proper control of this factor is of paramount importance [4,5]. A wort with a high content of fermented extract, i.e., containing a large amount of sugars and amino acids with an appropriate balance available to yeast, will significantly influence the high yield of the beer product [5].

The color of wheat beers in Europe is measured in EBC (European Brewery Convention) units and ranges on average from 8 to 20 EBC units. The color variations among wheat beers result from the proportion of wheat malt in the grist and the kilning process of the malts used to produce wheat beers. The kilning process influences the content of Maillard compounds (reaction products between amino acids and reducing sugars), which are transferred to the wort and final product during mashing [3,6,7]. Light wheat malts are kilned at a maximum temperature of 80–82 °C, while dark wheat malts are kilned at 100–110 °C, which increases the content of, for example, melanoidins, which contribute to a darker color [8]. Wheat beers are also characterized by a higher opalescence, which also affects the color of the finished beer. The turbidity of beers depends on the intensity of low-molecular-weight protein compounds derived from wheat malt, which have a low tendency to settle to the bottom of the bottle and thus contribute to increased turbidity [9]. During mashing and boiling, protein–polyphenol complexes are also formed, which contribute to the possibility of sedimentation in the beer, also affecting its color [10].

The acidity of wheat beers is related to the biological activity of the yeast and the quality of the raw materials used in the mashing process. High acidity combined with a low pH (around 4.0–4.4) reduces the risk of microbial contamination of the finished product [4,11]. A low pH is associated with a high content of free hydrogen ions in the beer, which increases the perception of acidity [7,12].

An optimal carbon dioxide content in the beer causes carbonation in wheat beer and enhances the feeling of saturation and refreshment, while beers characterized by low carbonation are not accepted by consumers. Carbon dioxide, along with proteins derived from the malts used, is responsible for the formation of beer foam because its molecules are encapsulated in a protein–protein colloidal system [13].

The perception of bitterness in beers is determined primarily by iso-α-acids and bitter acids derived from hops. The cultivar and quality of hops, as well as boiling time, influence the bitterness content in the produced beer [10]. Substances contained in hop cones (essential oils, hard and soft resins, and polyphenols), most often added in amounts of 20–60 g∙L^−1^, are responsible for the bitterness and aroma of the finished product. Iso-α-acids inhibit the growth of Gram (+) bacteria and stabilize beer foam [8,10]. Among the chemical compounds found in beers and derived from hops, xanthohumol and isoxanthohumol can be distinguished, which have anti-inflammatory, antimutagenic, and sedative effects on the human body; Flavanols and catechins—antioxidant activity; quercetin and kaempferol, found in beer as glycosides, may inhibit the growth of cancer cells; ferulic acid, a compound with strong antioxidant activity, delays beer spoilage by preventing the decomposition of iso-α-acids [10].

The fundamental difference between barley and wheat beers is the raw material composition (the origin of the malt). Barley beers are clear, full-bodied, with distinctive malt and hop notes, and examples of beers produced in this style include Lager and Ale. Wheat beers are often hazy, with a fruity aftertaste, and significantly less hoppy, and examples include Witbier. The available literature lacks research on winter wheat cultivars in beer production, which constitutes a huge research gap. The research is part of the ‘farm to fork’ strategy and encompasses a series of agrotechnical studies conducted in both rigorous field experiments in southwestern Poland and commercial canopy experiments in southeastern Poland. The primary goal of all the studies was to optimize winter wheat production for use in the malting and brewing industries. Based on the already agrotechnical tests performed and the quality of the wheat grain of the analyzed cultivars and the malts obtained from them, it was found that the Elixer and Gimantis cultivars are predisposed to further research. The results presented in this article concern the final stage of the research: the production of beers from wheat grain obtained from the field trials. The hypothesis of this study assumed that wheat beer brewed from at least one of the wheat cultivars cultivated in the three-year field experiment would achieve high technological quality after malting and brewing. These studies, together with those already published (from experiences on commercial farms), will allow, on the one hand, to develop and implement recommendations (including the recommended cultivar and nitrogen fertilization level) for farmers who want to grow wheat for malt and beer in a temperate climate, and on the other hand, they provide practical information for brewers and maltsters on how to conduct technological processes using wheat grain to obtain high-quality malt and wheat beers. In summary, after conducting agrotechnical research and evaluating wheat grain and malt, two wheat cultivars—Elixer and Gimantis—are recommended for malting production. The research presented in this article will determine which of these cultivars will yield higher-quality wheat beer, allowing for comprehensive research on these cultivars.

## 2. Materials and Methods

### 2.1. Research Material and Location

In three growing seasons, 2020/2021, 2021/2022, and 2022/2023, four winter wheat cultivars—Elixer, Lawina, Gimantis and Rockefeller—were grown in field experiments in Jelcz-Laskowice (51°21′ N; 17°35′ W), belonging to the Department of Herbology and Agricultural Cultivation Techniques, Wrocław, Poland. The wheat was fertilized with nitrogen at a dose of 60 kg N·ha^−1^. The field experiment conditions were presented in the localities of Belcar and Gorzelany [14].

### 2.2. Field Experiment Conditions

Certified winter wheat seeds were sown in subsequent years of the study: on 23 October 2020, 29 October 2021 and 28 October 2022, grain was sown at a rate of 300 units·m^−2^. The area of one experimental plot was 11 m^−2^. Experiments with selected wheat cultivars were carried out with fertilization of 60 kg N ha^−1^ (30 kg N ha^−1^ + 20 kg N ha^−1^ + 10 kg N ha^−1^). Fertilization with mineral nitrogen (ammonium nitrate 34% N Pulan) of winter wheat was carried out three times in the BBCH 25–26 phase (beginning of spring vegetation), in the BBCH 28–30 phase (end of tillering) and in the BBCH 32–34 phase (stem shooting). Wheat grain was harvested in the subsequent years of the study at full harvest maturity (29 July 2021, 25 July 2022 and 16 August 2023). More information is presented in Belcar and Gorzelany [14]. The average hydrothermal coefficients characterizing the weather pattern in the individual analyzed years of the study are presented in Table 1.

### 2.3. Preparation of Malts

For the study, cleaned wheat grains with a grain size > 2.5 mm were used. They were spread on metal germination plates and soaked to 45% moisture in a climatic cabinet (atmospheric parameters: 90% relative humidity and temperature 15 ± 1 °C). After obtaining a satisfactory sprout length (after 5 days), further growth was inhibited by drying at 40–80 °C for a total of 23 h (15 h—40 °C, 3 h—50 °C, 3 h—65 °C, 2 h—80 °C), and then degerminated.

### 2.4. Wheat Beer Brewing Process

The prepared wheat beers were prepared using the American Wheat infusion method. The wheat beers were prepared from two types of malt: 50% was wheat malt (quality parameters of wheat malts in the publication by Belcar and Gorzelany [14]) obtained from field experiments (four analyzed winter wheat cultivars, three analyzed growing seasons) and 50% was commercial barley malt from the Viking Malt malthouse (quality parameters of barley malt: extract 84% d.m., color 3.5 EBC, protein content 11.2% d.m., friability 86%, FAN 150 mg·L^−1^, diastatic power 285 WK). The wort was prepared as follows: two types of malt and 3 L of water per kilogram of malt were added to a ROYAL RCBM-30CK brew kettle (at 80% process efficiency). The first stage involved mashing for 60 min at 67 °C (with intensive mixing for the first 30 min). The next stage involved mashing at 72 °C for 15 min. The next step was to raise the mashing temperature to 78 °C for 10 min.

The wort was then heated to 100 °C at a rate of 2 °C/1 min and boiled with hops for 60 min. During this time, freeze-dried Amarillo hops (α-acid content 9.9%) were added in three doses:

1. 0-min boil—3 g hops
2. 45-min boil—1.5 g
3. 60-min boil (aroma hopping)—3 g.

The wort was cooled for 30 min. After cooling, the wort had an extract of 12.0 °P. The wort was inoculated with *Saccharomyces cerevisae* Safale US-05 yeast (6 × 10^9^ g^−1^), previously rehydrated according to the manufacturer’s instructions (0.58 g d.m.∙L^−1^ wort) in 30-L fermentation tanks and fermented at 21 °C for 14 days. The beer was then bottled after adding a 0.3% sucrose solution in water for refermentation and carbonation. Physicochemical tests were conducted one month after bottling.

Twelve wheat beers were produced, marked as follows: E (Elixer variety), L (Lawin variety), G (Gimantis variety) and R (Rockefeller variety), as well as 1 (first), 2 (second) and 3 (third growing season).

### 2.5. Qualitative Analysis of Wheat Beers

Qualitative analyses were performed on the obtained wheat beers, which included characterizing the fermentation process in accordance with 9.4 EBC [15] by determining the apparent extract (AE), original extract (OE), and real extract (RE). The parameters defining yeast activity during fermentation—apparent degree of attenuation (AA) and real degree of attenuation (RA)—were determined using the following formulas [3,16]:

AA = (OE − AE)/OE × 100(1)

RA = (OE − RE)/OE × 100(2)

The prepared wheat beers were analyzed for carbon dioxide content according to Polish Standard PN-A-79093-6:2000 [17], bitterness content according to method 9.8 EBC [18], alcohol content according to method 9.2.3 EBC [19], pH according to method 9.35 EBC [20], color according to method 9.6 EBC [21], and titratable acidity of the wheat beers, which was determined by titrating the tested beer with 1 M NaOH to pH = 8.2 [22]. The energy value of wheat beers was calculated using the formula [4]:

[kcal∙100 mL^−1^] = (7 × A(%*v*/*v*)) + (4 × E_r_(%*v*/*v*) × ρ) (3)

where density (ρ), real extract content (E_r_), and alcohol content (A)

### 2.6. Statistical Analysis

The study was statistically analyzed to examine the effect of wheat cultivar and growing season on the quality parameters of wheat beers. The analysis was performed in Statistica 13.3. Two-factor analysis of variance (Tukey’s HSD test) was used with a significance level of α = 0.05. Results were presented as *n* = 3 ± SD. The grouping of the tested winter wheat cultivars based on the quality characteristics of the obtained wheat beers was performed using cluster analysis.

## 3. Results

### 3.1. Graining of Wheat Malts Used in Beer Production

**Figure 1:**
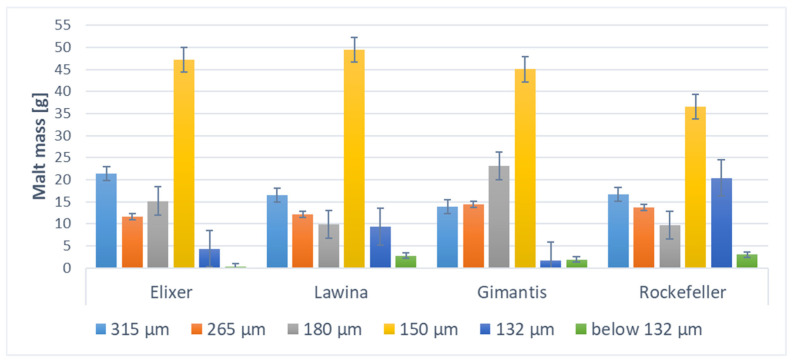
Granulometric composition of the analyzed wheat malt meal from which wheat beers were brewed.

The grinding process of wheat malt varied, similarly to milling into flour. This process was most difficult for the winter wheat cultivar Lawina. The granulometric distribution of the malt grist obtained from individual wheat cultivars varied depending on the cultivar analyzed. The malt grist obtained from the winter wheat cultivar Elixer was characterized by the highest proportion of the 1.8 and 1.0 mm fractions (both fractions accounting for over 80% of the total mass); the Lawina cultivar had the highest proportion of the 1.8 mm fraction (over 50% of the total mass), similar to the Gimantis cultivar, for which the 1.0 mm fraction accounted for approximately 30% of the total mass. In contrast, meal obtained from Rockefeller wheat grain was characterized by approximately 50% of the 1.0 mm fraction and over 35% of the 1.8 mm fraction. No differences were noted in the granulometric composition of the meals obtained from the grain of the analyzed wheat varieties from the individual years of the study (Figure 1).

### 3.2. Apparent and Real Extract Content

Malted wheat grain influences the efficiency of the fermentation process, the chemical composition of the beer wort, the alcohol content, and the real extract content, and consequently, the final quality of the beer product [7,23]. Apparent extract is the concentration of soluble compounds remaining in beer after fermentation [1]. The average apparent extract content in the analyzed wheat beers during the study years was 3.50% *m*/*m*, while the real extract content was 4.64% *m*/*m*, which was on average 20.45% higher than the apparent extract content (Table 2). The influence of the cultivar and growing season on the wheat beer parameters discussed was significant. E wheat beers (obtained from the Elixer cultivar) were characterized by the lowest average apparent extract content, while the highest apparent extract value was recorded for beers obtained from malt obtained from wheat grain of the Rockefeller cultivar (an increase of 33.18%). Wheat beers obtained from malt obtained from winter wheat of the Lawina cultivar had the lowest average real extract content, and among the analyzed wheat beers, those obtained from malt obtained from wheat of the Rockefeller and Gimantis cultivars had the highest real extract value; the analyzed parameter increased by 10.83% and 13.18%, respectively (compared to the beer product obtained from the raw material of the Lawina cultivar). The lowest difference between the average apparent and real extract values was obtained for wheat beers obtained from malt obtained from Rockefeller wheat grain (0.52%). The average apparent extract value in the obtained wheat beers varied between the individual growing seasons; the highest value was obtained in the second year of the study (3.70% *m*/*m*) and was on average 6.76% higher compared to the first year and 9.46% higher compared to the third year of the study. The varying mean values of real extract observed in the wheat beers obtained in the individual growing seasons were, like the mean value of apparent extract, dependent on the quality of the grain intended for malting, which was influenced by the meteorological conditions at the experimental site in subsequent years of the study. The highest mean value was obtained for the second year of the study (5.30% *m*/*m*), which was 17.17% higher compared to the first year and 20.38% higher compared to the third year of the study. However, the results obtained in the first and third years of the study did not differ.

### 3.3. Basic Wort Extract Content

**Figure 2:**
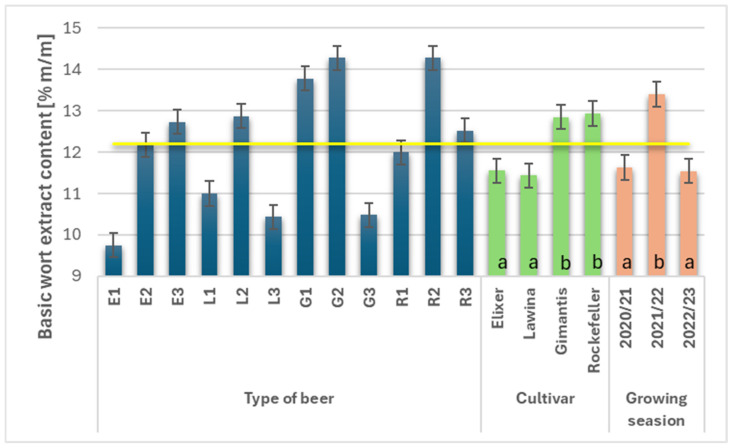
Basic wort extract content (% *m*/*m*) in the obtained wheat beers (beer E1, L1, G1, R1—first year of field research; beer E2, L2, G2, R2—second year of field research; beer E3, L3, G3, R3—third year of field research); a,b—statistically significant differences within the experimental factor at the confidence level of *p* = 0.05; the yellow line on the graph indicates the average value.

The average basic wort extract content in the wheat beers analyzed in the 2020–2023 study was 12.19% *m*/*m* (Figure 2) and varied depending on the cultivar and growing season. Wheat beers made from malt obtained from winter wheat of the Lawina and Elixer cultivars were characterized by a lower average wort extract content, amounting to 11.43% *m*/*m* and 11.55% *m*/*m*, respectively. Significantly higher wort extract values were recorded for beers made from malt obtained from wheat of the Rockefeller and Gimantis cultivars; the average increase in the analyzed parameter was 11.60% and 11.05%, respectively, compared to beer made from malt obtained from the Lawina cultivar. Differing meteorological conditions in individual growing seasons influenced grain parameters (obtained malt), and consequently, the average wort extract value in the resulting wheat beers. The highest value was obtained for the second year of the study (13.40% *m*/*m*), which was 13.21% higher compared to the first and 13.88% higher compared to the third year of the study.

### 3.4. Apparent and Real Attenuation

Attenuation of wheat beers is a parameter that describes the degree of conversion of sugars contained in the wort by brewer’s yeast into ethyl alcohol and carbon dioxide (alcoholic fermentation, [16]). The apparent and real attenuation values describe the dynamics of the fermentation process of a beer product [3]. The average apparent degree of attenuation in the analyzed wheat beers in the 2020–2023 study years was 71.13%, while the average real attenuation in the analyzed wheat beers in the study years was 62.11%, which was 12.68% lower than the average apparent attenuation (Table 3). The apparent attenuation in the beers was influenced by the cultivars used, but no effect of the growing season was observed, and the real attenuation was not affected by either the cultivar or the growing season. Wheat beers made from malt obtained from Rockefeller winter wheat were characterized by the lowest average apparent attenuation (67.47%). Significantly higher apparent attenuation values were characterized by beers obtained from malt obtained from wheat grain of the Elixer cultivar (74.59%) and Lawina cultivar (72.89%). Wheat beers obtained from malt obtained from winter wheat grain of the Elixer cultivar were characterized by the lowest average real attenuation value (60.89%), while among the analyzed wheat beers, the highest real attenuation degree was characterized by beers obtained from malt obtained from wheat grain of the Rockefeller and Lawina cultivars; their values were 62.99% and 62.69%, respectively. The lowest difference between the average apparent and real attenuation values was obtained for wheat beers produced from malt obtained from Rockefeller wheat grain (4.48%). The average apparent attenuation degree in the obtained wheat beers did not change across the individual growing seasons; the highest value was obtained in the second year of the study and it amounted to 72.78%. The highest value of real attenuation was obtained in the third year of the study and it amounted to 63.54%.

### 3.5. Ethanol Content

**Figure 3:**
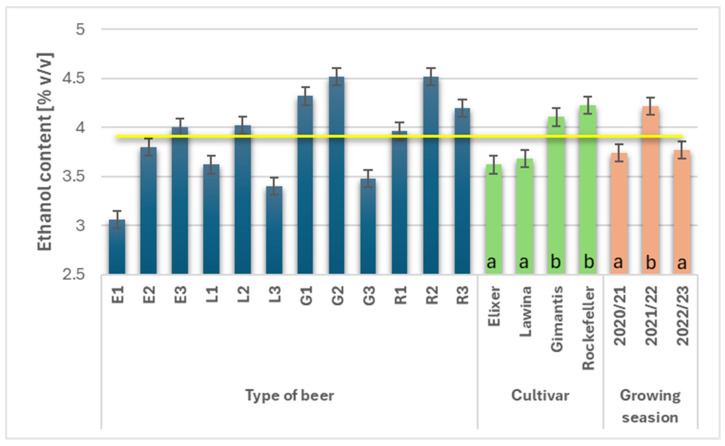
Ethyl alcohol content (% *v*/*v*) in the obtained wheat beers (beer E1, L1, G1, R1—first year of field research; beer E2, L2, G2, R2—second year of field research; beer E3, L3, G3, R3—third year of field research); a,b—statistically significant differences within the experimental factor at the confidence level of *p* = 0.05; the yellow line on the graph indicates the average value.

Ethanol is the primary product of the fermentation process, responsible for its sensory qualities, but it is also a distinguishing factor that determines the economics of beer production and the tax rate. In alcoholic fermentation, the main product is ethyl alcohol, but also, to a much lower extent, organic acids such as acetic acid and citric acid, which lower the pH of the beer product [3]. The average ethanol content in the analyzed wheat beers during the study years was 3.91% *v/v* (Figure 3). The cultivar and growing season had a significant impact on the ethanol content in beers obtained from the analyzed wheat malts. Wheat beers marked with the symbol E had the lowest average ethanol content (3.62% *v*/*v*), while wheat beers marked with the symbol L had a slightly higher ethanol content (3.68% *v*/*v*). Significantly higher average ethanol content was observed in beers obtained from malt obtained from wheat grain of the Rockefeller and Gimantis cultivars, and the increase in the analyzed parameter was, respectively, 14.42% and 11.92%. The average ethanol content in the obtained wheat beers varied in the individual growing seasons; the highest value was obtained for the second year of the study (4.22% *v*/*v*) and was 11.37% higher compared to the first and 10.66% higher compared to the third year of the study, while the results obtained in the first and third years of the study were not different.

### 3.6. Color of Wheat Beers

**Figure 4:**
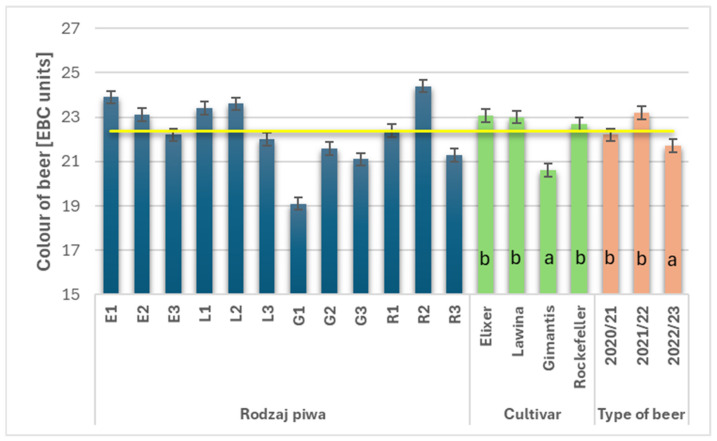
Color (EBC units) of the wheat beers obtained in subsequent years of the study (beer E1, L1, G1, R1—first year of field research; beer E2, L2, G2, R2—second year of field research; beer E3, L3, G3, R3—third year of field research); a,b—statistically significant differences within the experimental factor at the confidence level of *p* = 0.05; the yellow line on the graph indicates the average value.

**Figure A1:**
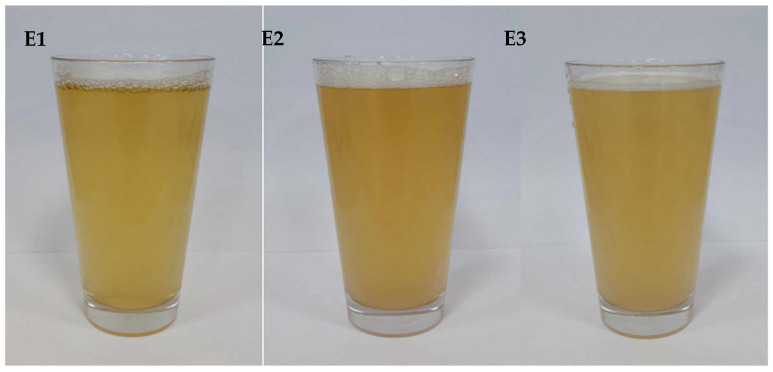
Appearance of wheat beers obtained from malt produced from Elixer wheat grain in subsequent years of field research (from the left: beer E1—first year; beer E2—second year; beer E3—third year).

**Figure A2:**
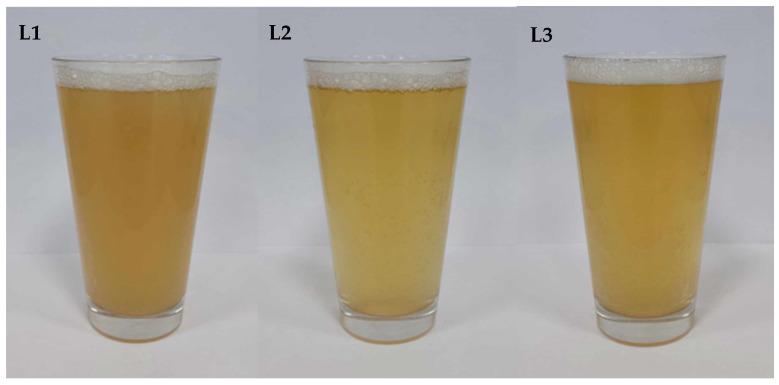
Appearance of wheat beers obtained from malt produced from Lawina wheat grain in subsequent years of field research (from the left: beer L1—first year; beer L2—second year; beer L3—third year).

**Figure A3:**
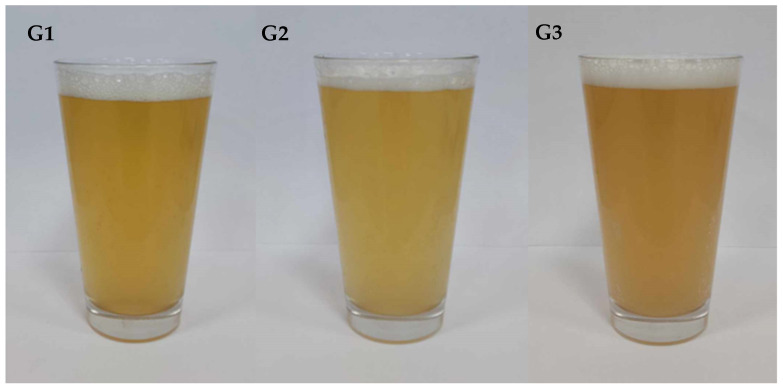
Appearance of wheat beers obtained from malt produced from Gimantis wheat grain in subsequent years of field research (from the left: beer G1—first year; beer G2—second year; beer G3—third year).

**Figure A4:**
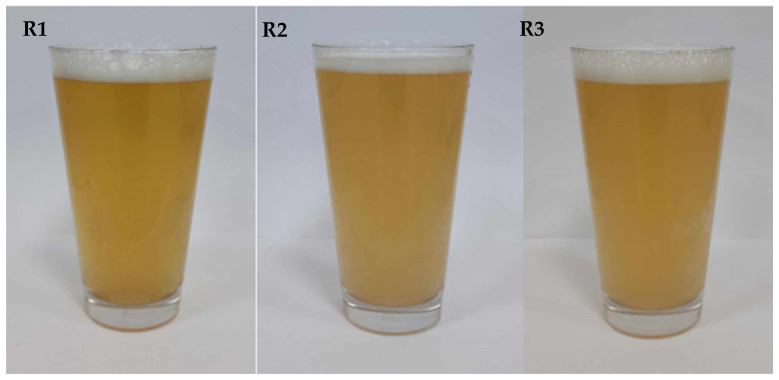
Appearance of wheat beers obtained from malt produced from Rockefeller wheat grain in subsequent years of field trials (from the left: beer R1—first year; beer R2—second year; beer R3—third year).

The average color value was 22.3 EBC units (Figure 4; Figure A1, Figure A2, Figure A3 and Figure A4—in the Appendix A). The cultivar and growing season had a significant effect on the color value. Wheat beers marked with the symbol G had the lowest average color value and were significantly lighter than the other wheat beers analyzed. The average color value in the obtained wheat beers varied depending on the growing season; the highest value was in the second year of the study (23.2 EBC units) and was 6.47% higher compared to the third year of the study, while the results obtained in the first and second years of the study did not differ.

### 3.7. Acidity and pH of Wheat Beers

**Figure 5:**
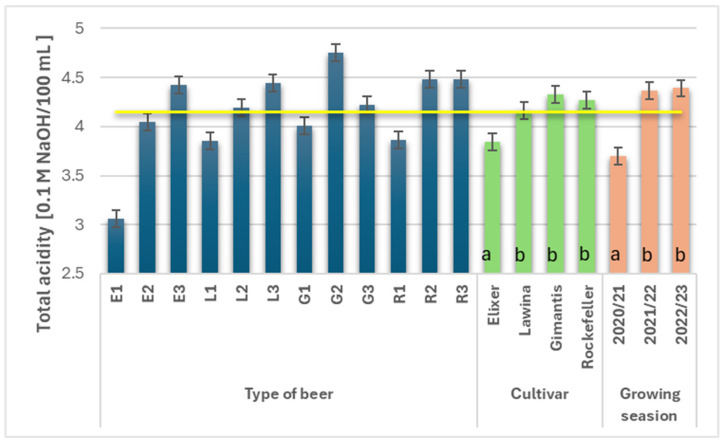
Total acidity (0.1 M NaOH·100 mL^−1^) of the obtained wheat beers (beer E1, L1, G1, R1—first year of field research; beer E2, L2, G2, R2—second year of field research; beer E3, L3, G3, R3—third year of field research); a,b—statistically significant differences within the experimental factor at the confidence level of *p* = 0.05; the yellow line on the graph indicates the average value.

The average acidity in the analyzed wheat beers during the study years was 4.15 (Figure 5). The cultivar and growing season had a significant impact on the acidity value of the beers. Wheat beers marked with the symbol E had significantly lower acidity (3.84), while the remaining beers had an average acidity of 4.25. The average acidity value in the individual growing seasons varied; the highest value was in the third year of the study (4.39) and was 15.72% higher compared to the first year of the study, while the results obtained in the second and third years of the study did not differ.

**Figure 6:**
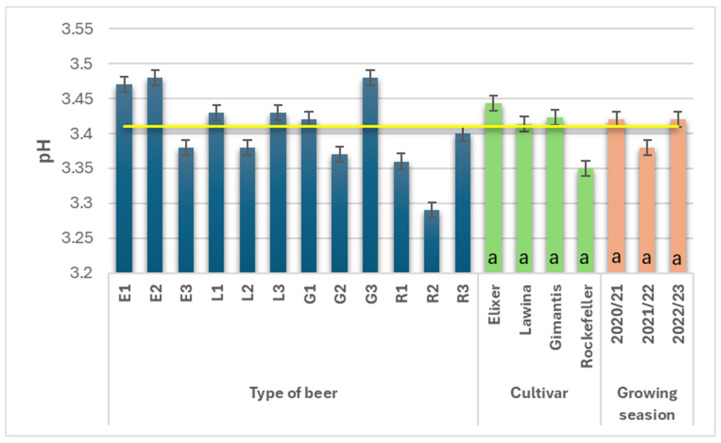
pH of the obtained wheat beers in subsequent years of the study (beer E1, L1, G1, R1—first year of field research; beer E2, L2, G2, R2—second year of field research; beer E3, L3, G3, R3—third year of field research); a—statistically significant differences within the experimental factor at the confidence level of *p* = 0.05; the yellow line on the graph indicates the average value.

The average pH value in the analyzed wheat beers during the study years was 3.41 (Figure 6). The pH value in the beers obtained from the analyzed wheat malts did not depend on the cultivar used or the growing season. Wheat beers obtained from malt obtained from Rockefeller winter wheat had the lowest average pH value (3.35) among the analyzed wheat beers, but these differences were insignificant. The average pH value in the obtained wheat beers did not vary across the growing seasons; the highest value was obtained for the second year of the study and was 3.38.

### 3.8. Bitterness Content

**Figure 7:**
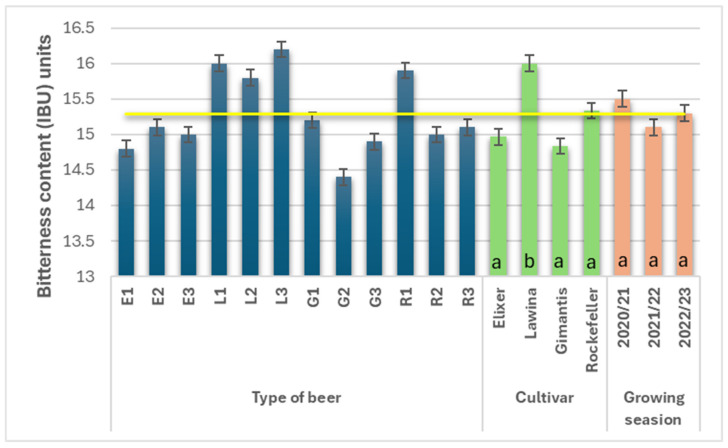
Bitterness content (IBU units) in the obtained wheat beers (beer E1, L1, G1, R1—first year of field research; beer E2, L2, G2, R2—second year of field research; beer E3, L3, G3, R3—third year of field research); a,b—statistically significant differences within the experimental factor at the confidence level of *p* = 0.05; the yellow line on the graph indicates the average value.

The perception of bitterness in beers, which causes an intense, bitter flavor, is determined mainly by iso-α-acids and bitter acids derived from hops. The cultivar and quality of hops, as well as the boiling time, influence the bitterness content in the produced beer [10]. Furthermore, the value of this parameter is influenced by the degree of protein reaction with polyphenols contained in malt [16]. The bitter taste of the finished beer product is partially neutralized by the sugars contained in the beer, the concentration of which contributes to the pleasant taste of beer products for the consumer and also influences the stabilization of beer foam [10]. The average bitterness content in the wheat beers analyzed during the study years was 15.3 IBUs (Figure 7), and its value was influenced by the malt cultivar used. Wheat beers marked with the G symbol were characterized by the lowest average bitterness content (14.8 IBU units), while those marked with the L symbol were characterized by a significantly higher bitterness content (16.0 IBU units; average bitterness content higher by 7.50%). The average bitterness content in the obtained wheat beers did not vary across the growing seasons; the highest value was obtained for the first year of the study and amounted to 15.5 IBU units. The addition of freeze-dried hops in the prepared and analyzed wheat beers was the same; the differences in bitterness perception were mainly caused by the addition of grain of the analyzed varieties used in the production of wheat malt, which served as a raw material in the mashing process.

### 3.9. CO_2_ Content

**Figure 8:**
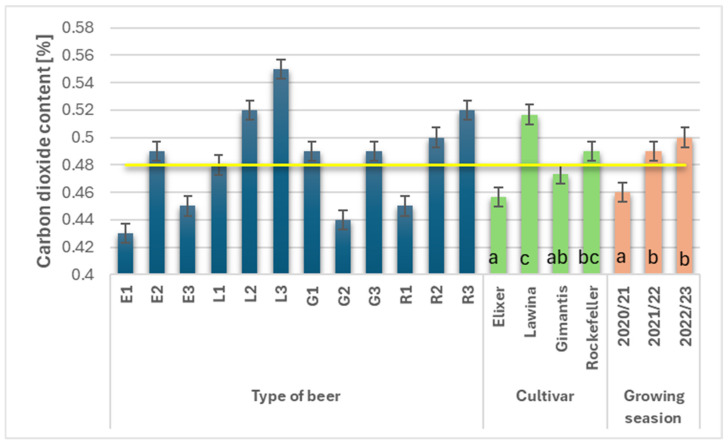
CO_2_ content (%) in the obtained wheat beers (beer E1, L1, G1, R1—first year of field research; beer E2, L2, G2, R2—second year of field research; beer E3, L3, G3, R3—third year of field research); a–c—statistically significant differences within the experimental factor at the confidence level of *p* = 0.05; the yellow line on the graph indicates the average value.

Carbon dioxide content in wheat beers is at an optimal level when it produces adequate carbonation, thereby enhancing the feeling of saturation and refreshment. In craft wheat beers, this value depends on the selection of the appropriate yeast strain, but also on the proportion of sugar (e.g., sucrose) added during the refermentation process. The higher the proportion of this latter ingredient, the higher the CO_2_ saturation of the finished beer product, but also the higher the proportion of residual sugars and glycerol. Glycerol is produced by yeast to limit dehydration, thus giving the beer a full and full-bodied flavor. However, unfavorable environmental conditions affect yeast metabolism, causing excessive glycerol production, which negatively impacts the carbon dioxide content in the finished beer product [24,25]. The cultivar and growing season significantly influenced the carbon dioxide content in wheat beers, averaging 0.48% (Figure 8). The lowest average carbon dioxide content (0.46%) was found in wheat beers marked with the symbol E, while significantly higher CO_2_ contents were recorded for beers obtained from malt obtained from wheat grain of the Rockefeller and Lawina cultivars; the average CO_2_ increase was 6.12% and 11.54%, respectively. The CO_2_ content in wheat beers was generally not significantly different in the individual growing seasons; the highest value was obtained in the third year of the study (0.50%) and it was 8.00% higher compared to the first year of the study, and the results obtained in the second and third years of the study did not differ significantly.

### 3.10. Energy Value of Wheat Beers

**Figure 9:**
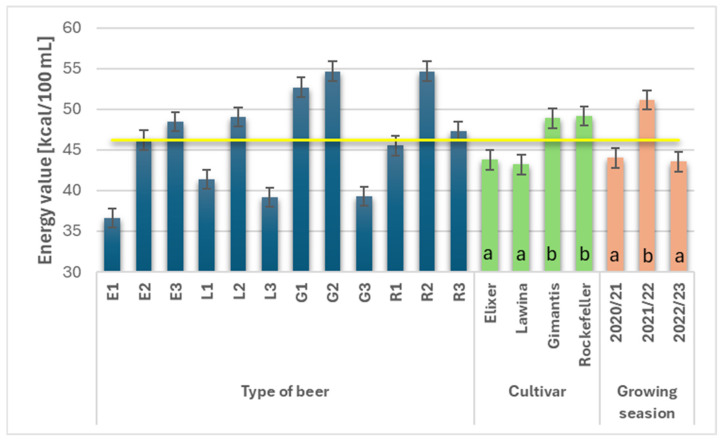
Energy value (kcal∙100 mL^−1^) of the obtained wheat beers (beer E1, L1, G1, R1—first year of field research; beer E2, L2, G2, R2—second year of field research; beer E3, L3, G3, R3—third year of field research); a,b—statistically significant differences within the experimental factor at the confidence level of *p* = 0.05; the yellow line on the graph indicates the average value.

A lower ethyl alcohol content in wheat beers results in a lower energy value of the resulting beer product [26]. The cultivar and growing season significantly influenced the energy value of wheat beers, with the average value being 46.24 kcal∙100 mL^−1^ (Figure 9). Wheat beers marked with the symbol L were characterized by the lowest average energy value (43.19 kcal∙100 mL^−1^), with a slightly higher caloric value (43.76 kcal∙100 mL^−1^) noted for beers obtained from malt obtained from wheat grain of the Elixer cultivar (insignificant differences). Significantly higher average energy values were observed in beers obtained from malt obtained from wheat grain of the Rockefeller and Gimantis cultivars; the analyzed parameter increased by 12.13% and 11.64%, respectively. The growing season significantly influenced the energy value of wheat beers, and the highest value was obtained in the second year of the study (51.14 kcal∙100 mL^−1^) and it was higher by 13.96% compared to the first and by 14.86% compared to the third year of the study, and the results obtained in the first and third year of the study did not differ significantly.

### 3.11. Cluster Analysis of the Obtained Wheat Beers

**Figure 10:**
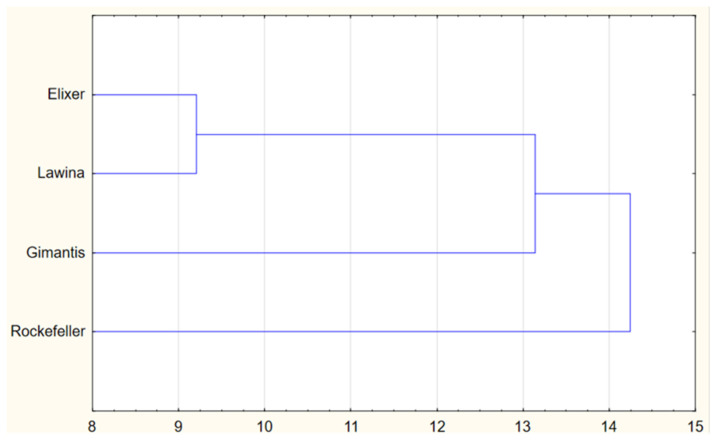
Dendrogram of similarity of winter wheat cultivars grown in field experiments in terms of beer quality parameters.

The aim of the cluster analysis was to group the analyzed winter wheat cultivars based on the quality characteristics of the resulting wheat beers (Figure 10).

Analysis of all quality characteristics of wheat beers obtained from malt derived from winter wheat grain grown in field experiments showed that the Elixer and Lawina cultivars (Cluster I) were characterized by comparable, but not the highest, quality of the resulting beers. In terms of the quality characteristics of wheat beers important from a brewer’s perspective, such as real extract content and ethanol content, the highest quality parameters were observed in beers brewed from malt derived from winter wheat grain of the Gimantis and Rockefeller cultivars, while the highest degree of real attenuation was observed in beers brewed from malt derived from Rockefeller grain. The clustering of the analyzed parameters for the Rockefeller and Gimantis cultivars was similar. The quality of the produced beers is influenced by the quality of the grain and malt, which are the main raw materials for their production. The quality of the raw materials produced in the analyzed years was influenced by weather conditions during the growing season, which varied between growing seasons, particularly during the shoot, earing, and ripening phases of wheat grain. These factors significantly influenced the quality of beers brewed with malt obtained from the analyzed winter wheat cultivars, which were characterized by high variability. The Gimantis cultivar, and to a lesser extent the Rockefeller cultivar, showed the least variability in quality among wheat beers brewed with malt obtained from grain obtained from three growing seasons in the Lower Silesian Voivodeship.

## 4. Discussion

Cereal grains characterized by high starch content are most often used for beer production. The malting process significantly affects the hydrolysis of the starch contained in the grain through the action of amylolytic enzymes, which yield fermentable sugars used in the yeast fermentation process [27]. Additives used in the raw material grist, including unmalted grain such as wheat, require modifications to the mashing process, which affects the flavor and aroma of the finished product [7]. Due to the properties of wort obtained exclusively from wheat malt (including filtration difficulties, long saccharification time, high viscosity), a mixture of barley and wheat malts, most often in amounts ranging from 30 to 60% *w*/*w*, is used for the production of wheat beers [28].

Wheat beers brewed in the American Wheat style should generally contain 50% of the grist, which was achieved in this study. These beers are characterized by a base wort extract content of 10–13.5% *m*/*m* and a real extract content of 1–3.5% *m*/*m*. The wheat beers produced in this study had a standard base wort extract content, indicating a properly brewed beer, while the average real extract was approximately 2% higher than the average standard extract content for beers brewed in the American Wheat style [16]. A higher real extract indicates incomplete fermentation, as the resulting beer still contains substrates, i.e., sugars, that could be converted by yeast into ethyl alcohol. This statement is supported by the results regarding ethanol obtained in the wheat beers analyzed in this study, which for the Elixer and Lawina cultivars was below the standard for the American Wheat style (4.0–5.5% *v*/*v*), while for the remaining analyzed wheat cultivars it was at the lower limit, which also influenced the attenuation of the produced wheat beers. Compared to the studies of other authors [3,16,26,29,30,31,32,33,34,35,36,37], OE, RE, AE and AA in the analyzed wheat beers were at a similar level, while RA and ethanol content were significantly lower. This could be largely due to the efficiency of the mashing process in the new brewing equipment (lower transfer of chemical compounds contained in the malt to the wort), and the low attenuation level in wheat beers results in a reduced ethyl alcohol content in the finished beer product.

According to the requirements for American Wheat beers, the color of the final product should be light, straw-colored, and range from 4 to 20 EBC units. The average color of the resulting wheat beers was 22.3 EBC units, which was above average; only the Gimantis cultivar achieved lower results. Compared to the studies of other authors [3,7,8,9,11,16,25,26,29,30,31,32,34,36,37], the color in the analyzed wheat beers was at a higher level, while in comparison to our previous studies, it was at the same level. Wheat beers not subjected to the filtration process are characterized by increased turbidity [11]. Grain subjected to the malting and drying process contains high levels of Maillard compounds, such as melanoidins, which are transferred to the beer during the brewing process and contribute to its darker color [3,7,16,24], and these compounds remain unchanged even by storage time [24]. The mashing and boiling process causes reactions between protein compounds and polyphenols, which can lead to the formation of sediments in the beer, also affecting its color [10]. Differences in the color of beers allow us to determine the intensity of the aging process [24]. Higher temperature and extended storage time of fresh wheat beer increase color intensity. This may be related to the gradual oxidation of polyphenols occurring in the finished beer product, and their higher content also accelerates the increase in color intensity [24]. The obtained fresh wheat beers were characterized by high turbidity, and this parameter decreased with extended storage time. The same relationship was observed in the study by He et al. [24]. This is related to the settling of brewer’s yeast cells at the bottom of the bottle. Furthermore, colloidal substances (including polysaccharides and high-molecular-weight proteins) also precipitate during storage. Storing commercial wheat beers at 20 °C did not increase their turbidity, while lowering this temperature to 5 °C (for 7 of 10 analyzed samples) significantly increased the turbidity of wheat beers during storage and simultaneously extended their shelf life [36].

The acidity of wheat beers is influenced by the activity of the yeast strain used and the quality of the raw materials. High acidity in beers negatively affects the conditions of the fermentation process, i.e., it can limit the fermentation efficiency of the yeast strain used and also increase the risk of microbiological contamination [11]. The higher acidity of wheat beers is also influenced by the proportion of wheat malt in the raw material [37]. The research results discussed in this paper are generally higher than those reported by other authors [9,11,16,26,29,30,31,32,34,35], with the exception of the study conducted by Byeon et al. [16].

The pH of wheat beers depends on the raw materials used; the presence of wheat malt lowers this parameter compared to beers made from unmalted wheat grain [37]. A low pH creates an unfriendly environment caused by the high content of free hydrogen ions in the beer product, which inhibits the growth of undesirable microflora, stabilizing the microbiological quality of the finished beer product [4,11], but also increases the perception of acidity [7,12]. Changes in the pH and acidity of the resulting wheat beers may indicate unfavorable and undesirable qualitative changes in the final product of the fermentation process. The research results discussed in this paper are generally lower than those reported by other authors [9,11,16,26,29,30,31,32,33,34,35].

Wheat beers brewed in the American Wheat style should have an IBU range of 15–30 units. Hopping can be done with European or American aromatic varieties. In this study, the American Amarillo variety was used. Bitterness in this style of wheat beers should not be excessively high. Subsequent hop additions can be larger (especially those added for aroma after boiling or during wort cooling). The wheat beers produced in this study were characterized by a low bitterness content, which may be acceptable to consumers, especially women, who generally prefer milder beers.

American wheat is characterized by a low to medium full-bodied flavor. Carbonation is typically medium to high. It is smooth and crisp. Without harshness, astringency, or stickiness, it is very drinkable and refreshing. Carbon dioxide content depends on the course of alcoholic fermentation. By adding the same amount of sugar as a starting dose to stimulate yeast activity in each study, the potential of individual malts obtained from the analyzed winter wheat varieties can be determined, as the course of the fermentation process and, consequently, the content of individual products of this process, i.e., ethanol and carbon dioxide, depends on the sugars contained in their grain and their availability. The wheat beers discussed were characterized by a very similar carbon dioxide content (0.45% on average), regardless of the analyzed cultivar and growing season.

The wheat beers analyzed in this article were characterized by a lower caloric content compared to previous studies, primarily due to their lower ethanol content. On the one hand, a lower ethanol content, given the same fermentation method, may indicate potential production errors. However, from a nutritional perspective, this is a positive aspect, as it provides the body with a lower dose of harmful substances and a higher dose of substances with health benefits, such as polyphenols and vitamins. Sensory impressions are a crucial attribute of beer quality. The American Wheat style in which the wheat beers in question were produced should be characterized by malty and grainy aromas, a feeling of freshness and full flavor with a slight intensity.

## 5. Conclusions

The quality of wheat beers obtained from the analyzed winter wheat cultivars produced in three growing seasons was found to be varied. Wheat beers obtained from malt of the Rockefeller and Gimantis cultivars were characterized by the highest real extract values, ethyl alcohol content, and therefore, caloric content of the finished beer product. Furthermore, wheat beers obtained from malt from the Gimantis cultivar were characterized by the lightest color and the lowest bitterness. The highest quality of wheat beers was obtained from grain from the second growing season, regardless of the cultivar used. At a fertilization rate of 60 kg N∙ha^−1^, the Gimantis variety is characterized by the smallest fluctuations in the quality of the finished product in relation to the growing season and is dedicated as a wheat variety that can be recommended for the brewing industry. By implementing the “from farm to fork” concept, this research will soon allow for the combination of agronomic guidelines for farmers, and maltsters and brewers will receive full information on the technological parameters of wheat beer production from Gimantis wheat.

## Figures and Tables

**Table 1:** Sielianinow’s hydrothermal coefficient k during the plant vegetation period in the analyzed years of the study.

Growing Season	Month												
	09	10	11	12	01	02	03	04	05	06	07	08	Average
2020/21	2.07	1.31	1.41	5.03	2.16	4.44	1.10	0.20	1.30	0.60	0.45	1.13	1.77
2021/22	0.05	0.28	2.52	2.22	0.34	0.14	0.23	1.30	1.33	0.02	1.53	6.05	1.33
2022/23	6.65	3.57	0.75	1.50	2.47	5.15	1.72	1.81	0.64	0.82	0.32	1.65	2.25
2008–2019	1.16	1.24	1.55	0.49	5.13	1.03	1.91	0.87	1.08	1.25	1.24	0.81	1.48

**Table 2:** Apparent and real extract content (% *m*/*m*) in the obtained wheat.

Growing Season	Cultivar	Apparent Extract Content [%]	Real Extract Content [%]
2020/21	Elixer	3.31 ± 4.51	3.75 ± 5.32
	Lawina	2.84 ± 4.03	3.95 ± 2.56
	Gimantis	3.56 ± 3.82	5.49 ± 4.53
	Rockefeller	4.09 ± 3.16	4.37 ± 2.62
2021/22	Elixer	2.44 ± 4.21	4.81 ± 4.68
	Lawina	3.06 ± 3.56	5.12 ± 4.07
	Gimantis	4.26 ± 3.88	5.63 ± 4.01
	Rockefeller	5.03 ± 4.05	5.63 ± 3.11
2022/23	Elixer	2.83 ± 3.11	5.01 ± 4.27
	Lawina	3.31 ± 3.18	3.78 ± 2.19
	Gimantis	3.54 ± 2.53	3.68 ± 2.52
	Rockefeller	3.73 ± 5.14	4.40 ± 3.21
**Average for cultivation**	Elixer	2.86 ^a^ ± 2.77	4.52 ^b^ ± 3.89
	Lawina	3.07 ^b^ ± 3.12	4.28 ^a^ ± 2.12
	Gimantis	3.79 ^c^ ± 1.89	4.93 ^c^ ± 3.18
	Rockefeller	4.28 ^d^ ± 2.79	4.81 ^c^ ± 1.66
**Average for cultivation**	2020/21	3.45 ^a^ ± 1.57	4.39 ^a^ ± 2.61
	2021/22	3.70 ^b^ ± 2.56	5.30 ^b^ ± 2.11
	2022/23	3.35 ^a^ ± 3.61	4.22 ^a^ ± 2.51

^a–d^—statistically significant differences within the experimental factor at the confidence level of *p* = 0.05.

**Table 3:** Apparent and real attenuation (%) in the obtained wheat beers.

Growing Season	Cultivar	Apparent Attenuation [%]	Real Attenuation [%]
2020/21	Elixer	66.05 ± 4.51	61.54 ± 5.32
	Lawina	74.18 ± 4.03	64.09 ± 2.56
	Gimantis	74.17 ± 3.82	60.16 ± 4.53
	Rockefeller	65.89 ± 3.16	63.55 ± 2.62
2021/22	Elixer	79.95 ± 4.21	60.48 ± 4.68
	Lawina	76.22 ± 3.56	60.22 ± 4.07
	Gimantis	70.17 ± 3.88	60.57 ± 4.01
	Rockefeller	64.78 ± 4.05	60.57 ± 3.11
2022/23	Elixer	77.77 ± 3.11	60.04 ± 4.27
	Lawina	68.26 ± 3.18	63.76 ± 2.19
	Gimantis	64.41 ± 2.53	64.89 ± 2.52
	Rockefeller	71.73 ± 5.14	64.86 ± 3.21
**Average for cultivation**	Elixer	74.59 ^c^ ± 2.77	60.89 ^a^ ± 3.89
	Lawina	72.89 ^bc^ ± 3.12	62.69 ^a^ ± 2.12
	Gimantis	69.58 ^ab^ ± 1.89	61.87 ^a^ ± 3.18
	Rockefeller	67.47 ^a^ ± 2.79	62.99 ^a^ ± 1.66
**Average for cultivation**	2020/21	70.07 ^a^ ± 1.57	62.34 ^a^ ± 2.61
	2021/22	72.78 ^a^ ± 2.56	60.46 ^a^ ± 2.11
	2022/23	70.54 ^a^ ± 3.61	63.54 ^a^ ± 2.51

^a–c^—statistically significant differences within the experimental factor at the confidence level of *p* = 0.05.

## Data Availability

The original contributions presented in this study are included in the article. Further inquiries can be directed to the corresponding author.
